# Effects of 18 Months of Growth Hormone Replacement Therapy on Bone Mineral Density in Patients with Adult Growth Hormone Deficiency: A Retrospective Study

**DOI:** 10.1155/2023/4988473

**Published:** 2023-03-31

**Authors:** Ya-Yin Shen, Jia-Ni Ma, Zi-Yu Ren, Jie Liu, Xin-Yi Zhou, Xue-Rui Xie, Wei Ren

**Affiliations:** ^1^Department of Endocrinology, The First Affiliated Hospital of Chongqing Medical University, Chongqing, China; ^2^Department of Endocrinology, The Second Affiliated Hospital of Chongqing Medical University, Chongqing, China

## Abstract

**Objective:**

The effect of physiological dose growth hormone (GH) replacement therapy on bone mineral density (BMD) in adults with growth hormone deficiency (GHD) is not well defined. We aimed to investigate the effects of 18 months of treatment with recombinant human growth hormone (rhGH) at physiological doses on BMD, body composition (BC), and quality of life (QoL).

**Methods:**

Sixty-eight patients diagnosed with adult growth hormone deficiency (AGHD) in our hospital were included in this retrospective study. All patients received individualized rhGH replacement to maintain normal serum insulin-like growth factor-1 (IGF-1) levels. BMD and BC measurements were performed by dual energy X-ray absorptiometry (DXA). Excluding those with incomplete follow-up data, we analyzed BMD in 68 patients, as well as BC and QoL in 36 of them.

**Results:**

Compared with the baseline, lumbar spine BMD decreased by 0.008 g/cm^2^ (*P*=0.006) and increased by 0.011 g/cm^2^ (*P*=0.045) at month 18, and total hip BMD decreased by 0.005 g/cm^2^ (*P*=0.008) and did not change significantly from the baseline at month 18. The changes in BMD did not differ by sex, and the increase in BMD was more pronounced in patients with low *Z*-scores at the baseline (lumbar spine: *P*=0.005 and total hip: *P*=0.018). The percentage change from the baseline in BMD was greater for the lumbar spine than for the total hip (*P*=0.003). Lean body mass (LBM) increased significantly (*P*=0.012), total body fat ratio (TBF%) decreased significantly (*P*=0.011), visceral adipose tissue (VAT) decreased significantly (*P*=0.016), and QoL improved significantly (*P* < 0.001).

**Conclusions:**

Within 18 months of treatment, bone resorption manifested first, BMD decreased to a nadir at month 6, and then it increased. The increase in BMD was greater in the lumbar spine than in the hip, and the increase was more pronounced in patients with low BMD. Eighteen months of rhGH replacement therapy significantly improved lumbar spine BMD and improved BC and QoL.

## 1. Introduction

Adult growth hormone deficiency (AGHD) is a multifactorial group of endocrine disorders characterized by reduced bone mineral density (BMD) and lean body mass (LBM), increased visceral fat, insulin resistance, dyslipidemia, reduced muscle strength, and reduced quality of life (QoL) [1].

Conclusive evidence indicates that GH deficiency in adulthood results in decreased BMD, decreased bone turnover, and higher fracture risk rates [2]. Growth hormone (GH) exerts its effects mainly indirectly through insulin-like growth factor-1 (IGF-1), while GH itself can also act directly on different cells, leading to the growth of bones and different organs. GH and IGF-1 have important roles in maintaining bone metabolic homeostasis in humans [3]. The GH/IGF-1 axis promotes longitudinal bone growth, skeletal maturation, and bone mass acquisition during adolescence and acts to maintain bone mass in adults. The GH/IGF-1 axis exerts its anabolic effects on bone through multiple mechanisms, and it can stimulate the proliferation of bone marrow mesenchymal stem cells and promote their differentiation into osteoblasts. The GH/IGF-1 axis also maintains bone mass by maintaining a balance between osteoblasts and osteoclasts [4–6].

Several studies have shown that BMD is positively affected by rhGH replacement therapy in patients with AGHD. Several long-term studies [7–11], the longest of which, up to 15 years, have shown that rhGH replacement significantly improved BMD in patients with AGHD, with a peak bone mass in the seventh year followed by a downward trend [7]. However, in some short-term studies within 24 months, these findings have been inconsistent or even contradictory due to differences in the dose of GH, duration of treatment, study population, study methods, and equipment used to detect BMD [12–18]. Several findings suggest that during the first 12 months of rhGH replacement therapy, in which bone resorption predominates, no improvement in BMD is observed and even a downward trend [17, 19, 20]. The response of bone to GH is not the same in various parts of the body, and the changes observed in the long bones of the extremities, the lumbar spine, and the hip joints are inconsistent [16, 19]. The effect of GH on bone mass is different between patients with adult-onset GH deficiency (AO-GHD) and those with childhood-onset GH deficiency (CO-GHD) [21] because patients with CO-GHD may not have reached peak bone mass, and some may have used rhGH before, in some studies, the two groups were not distinguished. All of these factors have influenced the outcome of response to GH replacement. These studies were all conducted in Caucasians, and there were no relevant studies in Asians, so these conclusions do not serve as an absolute reference for the effects of rhGH treatment on BMD in Asian patients with AGHD.

Multiple studies have shown that replacement therapy with rhGH in patients with AGHD also produces some improvement in body composition and can reduce body fat mass (BFM), increase lean body mass [22, 23], and enhance patient quality of life [18, 24].

We conducted a retrospective study aimed at exploring the effects of short-term rhGH replacement on bone mineral density, body composition, and quality of life.

## 2. Data and Methods

### 2.1. Study Population

We selected 68 patients (22 males and 46 females; mean age 42.90 ± 11.52 years) with AGHD who visited in the Endocrinology Department of the First Affiliated Hospital of Chongqing Medical University from September 2018 to September 2022. AGHD diagnosis was based on the American College of Endocrinology recommendation of an insulin tolerance test (ITT) as the gold standard [25] and a peak growth hormone level <5.0 mg/L. They were all AO-GHD patients and had not received prior GH therapy. All were assessed for thyroid, gonadal, and adrenal function, and all patients with deficiencies of other hormones except for GH were on stable hormone replacement therapy for more than 6 months. The exclusion criteria were as follows: (1) CO-GHD; (2) known or suspected secondary osteoporosis; (3) antiosteoporotic drugs were used or have been clearly established to have an effect on BMD; (4) BMD was not reviewed at regular follow-up visits, and data were incomplete; (5) systolic blood pressure>140 mmHg or diastolic blood pressure >90 mmHg under treatment; (6) diabetes or poor fasting glucose control; (7) combined severe liver and kidney disease; (8) severe heart disease; and (9) present, past, or family history of malignancy. Finally, we selected 68 patients, all of whom had been on GH therapy for 18 months and had BMD and other indexes assessed at the baseline before GH treatment and at 6, 12, and 18 months, respectively. Only 36 subjects had body composition data available at the baseline and 18 months, so only 36 subjects were included in the comparison statistics of body composition and physical data (7 males and 29 females; mean age: 38.75 ± 8.75 years).

An individualized rhGH dosage regimen was used in all patients to maintain IGF-1 at normal levels. Patients were subcutaneously injected daily with rhGH (Jintrolong®, China) by using an automatic pen device. The initial dose was 0.5 IU/day, and in the first month after treatment initiation, doses were adjusted based on subject changes in efficacy and safety indicators and IGF-1 levels. Patients were followed up every 3 months with questionnaires, physical measurements, and blood biochemical measurements. Bone mineral density was measured every 6 months. The rhGH dose was adjusted according to the actual situation with each increase or decrease of 0.17 mg/day (0.5 IU/day), and the highest dose did not exceed 0.67 mg/day (2 IU/day).

All protocols were approved by the ethics committee of our hospital and conformed to the Declaration of Helsinki, and all subjects gave written informed consent.

### 2.2. General Clinical Information Collection and Anthropometric Measurements

All patients completed a uniformly formulated questionnaire, and clinical history taking and physical examinations were performed on all subjects by the same investigator. The anthropometric measurements of height, weight, waist circumference (WC), hip circumference (HC), grip strength, and blood pressure were measured as described in our previous study [22]. The body mass index (BMI) was calculated by weight (kg)/height^2^ (m^2^), and waist-hip ratio (WHR) was calculated by waist circumference (cm)/hip circumference (cm).

### 2.3. Blood Biochemical Measurements

All subjects had a light diet the previous night and fasted for 8–12 hours, and then blood was collected from the elbow vein the next morning and centrifuged to obtain fresh serum. Fasting blood glucose (FPG), fasting insulin (FINS), liver and kidney function, and blood lipid profiles were measured. GH, IGF-1, and thyroid function were also measured. FPG was measured using the glucose oxidase method (Biosen5030 rapid glucose detector, Necar, Germany). Lipid profiles were measured by a biochemical autoanalyzer (Olympus AU5400, Japan). FINS, GH, IGF-1, and thyroid function were measured by chemiluminescence (Chemi immuno luminescence kit, Roche).

### 2.4. Bone Mineral Density and Body Composition

BMD and BC measurements were performed by a dual energy X-ray absorptiometry (DXA) scanner (Hologic Discovery QDR® Series, USA). The subjects removed metals and jewelry, lying flat on the scanner, which was manipulated by the same professionally trained staff. Quality control assays for DXA were performed daily with a coefficient of variation (CV) <1.0%. BMD of the lumbar spine L2-L4 and total hip were measured, and *T*-scores and *Z*-scores were automatically generated using standard procedures and DXA reference database software as previously described [22]. To eliminate the influence of age on BMD, the reduction in BMD of the subjects was assessed with *Z*-scores. In this study, subjects with *Z* < −1 were classified into the low bone mass group, and subjects with *Z* ≥ −1 were classified into the high bone mass group. Body fat mass, lean body mass, total body fat ratio a (TBF%), and visceral adipose tissue (VAT) were also estimated by DXA. Total body lean/fat ratio was calculated by LBM (g)/BFM (g).

### 2.5. Quality of Life

Quality of Life-Assessment of Growth Hormone Deficiency in Adults (QoL-AGHDA) was used to assess the quality of life, a questionnaire specifically designed to assess the QoL of AGHD patients, an indicator used in daily monitoring or clinical trials of patients, and each language version has been shown to have good reliability and internal consistency [26, 27]. This questionnaire consists of a total of 25 individual questions, which are scored as 0 if the patient indicates the absence of this problem, 1 if the patient indicates the presence of a problem, and a high total score indicates poor quality of life.

### 2.6. Statistics

Statistical analysis was performed using SPSS 25.0 software. We performed the Kolmogorov–Smirnov test or the Shapiro–Wilk test a priori on the data of all subjects to determine whether they belonged to a normal distribution. Data are expressed as the mean ± standard deviation (SD) if they conformed to a normal distribution and as *M* (*P*25 and *P*75) if they did not conform. The comparisons of means between the groups that conformed to a normal distribution and homogeneity of variance were performed using the independent samples *t*-test, and those that were not consistent were performed by the Mann–Whitney *U* test. Differences in data at each time point of rhGH treatment were compared using the paired sample *t*-test or Wilcoxon test. Differences in change between BMD of the hip and lumbar spine were compared using the Wilcoxon test. The comparisons of differences in BMD change between the groups for sex and *Z*-scores were performed using the Mann–Whitney *U* test. The correlation analysis was performed using Pearson, Spearman's correlation analysis. *P* values <0.05 were considered significant.

## 3. Results

### 3.1. Baseline Characteristics

According to the inclusion criteria, a total of 68 subjects (22 males, 46 females; mean age 43.22 ± 11.32 years) were included in the study. The baseline characteristics of all patients are shown in Table 1. The males in this study had significantly greater WHR than females (*P* < 0.001), as well as the male subjects possessing lower lumbar spine BMD *T*-scores (*P* < 0.05) and *Z*-scores (*P* < 0.01). The peak GH after ITT correlated significantly with IGF-1 values (*r* = 0.531; *P* < 0.001). We categorized the baseline BMD and *Z*-score of all subjects into peak GH ≥ 3 and peak GH < 3 groups according to the GH peak, and we did not observe differences in BMD or *Z*-scores between the two groups. We also did not observe correlations between peak GH with baseline BMD or *Z*-scores.

### 3.2. Serum IGF-1 Concentration

**Figure 1:**
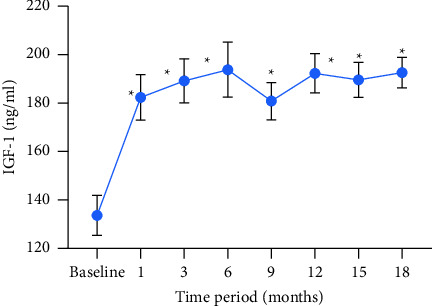
Serum IGF-1 changes over the 18-month treatment period. The results are presented as the mean ± SE. ^*∗*^*P* < 0.001 compared with the baseline (student's *t*-test).

Serum IGF-1 significantly increased after 18 months of rhGH replacement therapy compared to the baseline (*P* < 0.001). Serum IGF-1 was significantly elevated from the baseline at each follow-up visit, with the increase being most pronounced within the first month of treatment (Figure 1).

### 3.3. BMD

**Figure 2:**
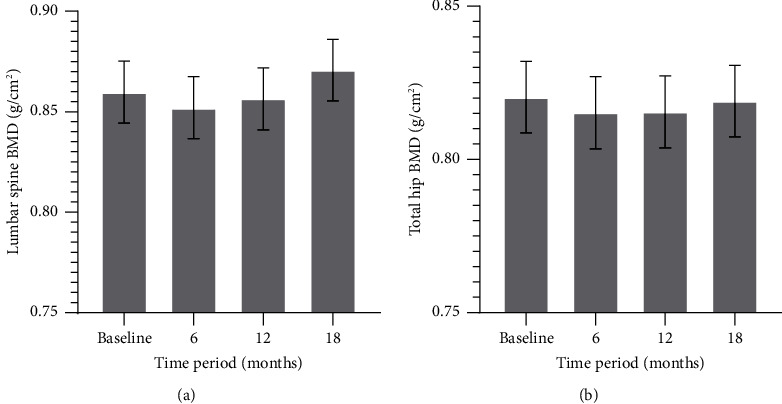
BMD changes over the 18-month treatment period. (a) Lumbar spine BMD; (b) total hip BMD. The results are presented as the mean ± SE.

BMD changes in the lumbar spine and total hip are shown in Table 2 and Figure 2. Compared with the baseline, BMD of the lumbar spine increased significantly after 18 months of rhGH therapy by 1.4% in percentage and by 0.011 g/cm^2^ in actual value (*P*=0.045). BMD decreased significantly at month 6, with a percentage decrease of 0.89% and an actual value of 0.008 g/cm^2^ (*P*=0.006). BMD was lower than the baseline at month 12, but the difference was not statistically significant (*P*=0.412).

Compared with the baseline, BMD of the total hip did not change significantly after 18 months (*P*=0.701) and decreased significantly at 6 months, with a percentage decrease of 0.62% and an actual value decrease of 0.005 g/cm^2^ (*P*=0.008). BMD began to increase after 6 months, by 0.46% at month 18 relative to that at month 6, with an actual increase of 0.0041 g/cm^2^; however, it was not significant (*P*=0.201), and the BMD of the hips remained lower than that at the baseline after 18 months of rhGH therapy, although the difference was not significant (Table 2).

**Figure 3:**
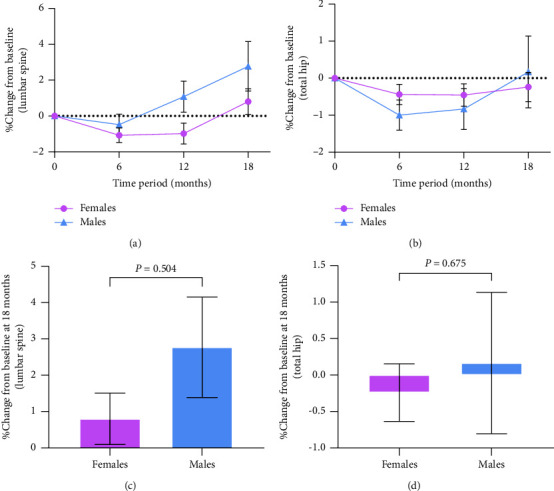
Differences between sexes in percentage change in BMD. (a) Changes in lumbar spine BMD over an 18-month treatment period; (b) changes in total hip BMD over an 18-month treatment period; (c) difference in change between sexes in lumbar spine BMD at the 18^th^ month; (d) difference in change between sexes in total hip BMD at the 18^th^ month. The results are presented as the mean ± SE. *P* values are not significant (Mann–Whitney *U* test).

**Figure 4:**
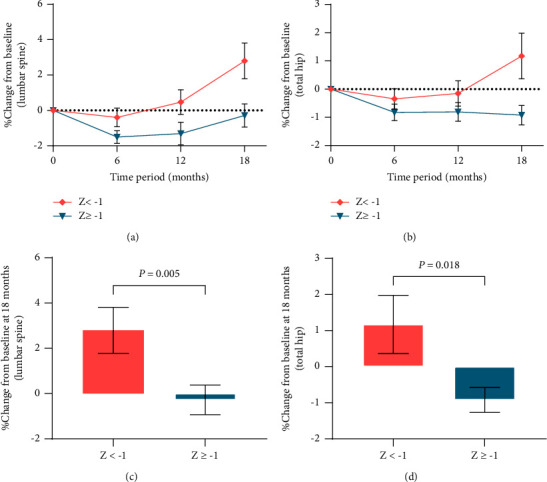
Differences between different *Z*-scores' groups in percentage change in BMD. (a) Changes in lumbar spine BMD over an 18-month treatment period; (b) changes in total hip BMD over an 18-month treatment period; (c) difference in change between different *Z*-scores' groups in lumbar spine BMD at the 18^th^ month. *P* < 0.01 for the *Z* *<* −1 group compared with the *Z* ≥ −1 group (Mann–Whitney *U* test); (d) difference in change between different *Z*-scores' groups in total hip BMD at the 18^th^ month. *P* < 0.05 for the *Z* *<* −1 group compared with the *Z* ≥ −1 group (Mann–Whitney *U* test). The results are presented as the mean ± SE.

**Figure 5:**
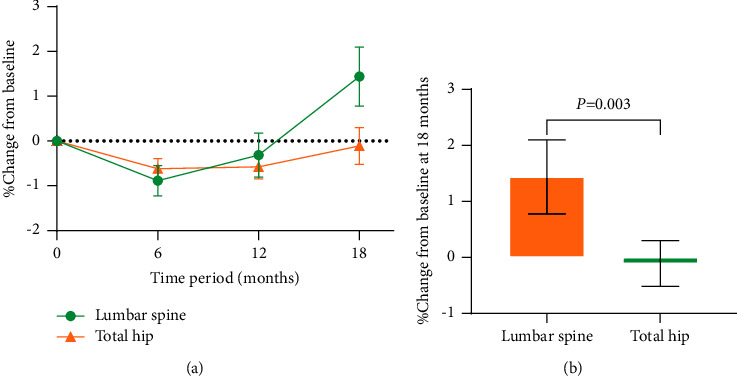
Differences in percentage change in BMD of the lumbar spine and total hip. (a) Changes in BMD of the lumbar spine and total hip over an 18-month treatment period; (b) difference in change between lumbar spine BMD and total hip BMD at the 18th month. *P* < 0.01 for the lumbar spine BMD compared with the total hip BMD (Wilcoxon test). The results are presented as the mean ± SE.

We performed post hoc stratification of BMD data, grouping BMD of the lumbar spine and total hip separately by sex (Figure 3) and *Z*-scores (Figure 4). We found that the percentage changes from the baseline in BMD at the lumbar spine and total hip did not differ by sex (Figure 3), but subjects with *Z* < −1 had a greater increase, and the changes were significantly greater than those with *Z* ≥ −1 (lumbar spine: *P*=0.005 and total hip: *P*=0.018) (Figure 4). We also found that the percentage change from the baseline in BMD at the lumbar spine was greater after 18 months of rhGH therapy than that at the total hip (*P*=0.003) (Figure 5).

We found no correlation between serum IGF-1 levels and BMD or *Z*-scores at any time point (data not shown in the article).

### 3.4. Body Composition, Physical Measurements, and QoL

As previously mentioned, only 36 subjects were included in the comparison statistics of body composition and physical data (Table 3). Compared with the baseline, no significant changes were observed in height, weight, BMI, WC, HC, and WHR at month 18, and BMI means before and after treatment were within the normal range (BMI < 24 kg/m^2^). BFM was reduced but not significantly (*P*=0.094), LBM was significantly increased (*P*=0.012), total body lean/fat ratio was significantly increased (*P*=0.003), TBF% was significantly decreased (*P*=0.011), and VAT was significantly decreased (*P*=0.016). It illustrates the increase in the proportion of lean mass, the decrease in the proportion of fat mass, and the unchanged body weight. Grip strength increased significantly (*P*=0.003). Diastolic blood pressure decreased significantly (*P*=0.036), and blood pressure was within normal limits before and after treatment. Qol-AGHDA scores decreased significantly (*P* < 0.001).

## 4. Discussion

In this retrospective study, we investigated the effects of rhGH replacement therapy at physiological doses on BMD in patients with AGHD, and we adjusted the dosage of rhGH by using the levels of serum IGF-1 as a reference index to ensure that the IGF-1 levels of the patients were below the upper limit of the normal range for the same age. Some previous studies may have employed low doses of rhGH replacement for observation, which may interfere with the results because of insufficient elevation of serum IGF-1 levels [18], and some studies used higher doses, resulting in a high incidence of side effects in patients [28]. Our individualized rhGH dose replacement is more clinically valuable in the treatment of patients with AGHD. Some of the previous studies included patients with CO-GHD, but their response to rhGH is not consistent with AO-GHD patients, thereby causing interference with the study results, and the two populations cannot be combined in such studies [20]. In order to exclude the confounding effect of CO-GHD on the study results, only patients with AO-GHD were included in this study.

Studies have shown that BMD in adults with GHD is reduced [2], there is no difference in BMD reduction between isolated GHD patients and multiple pituitary hormone deficiency patients [29], and there is no significant difference in BMD changes between the two populations after receiving rhGH replacement. This shows that the decrease in bone mass in patients with AGHD is mainly due to the lack of GH but is not related to other pituitary hormones. In our study, the mean T-score of BMD of all subjects at the baseline was within the range of osteopenia, indicating that the BMD of AGHD patients was lower than that of normal healthy people. Therefore, theoretically speaking, the supplementation of physiological dose of GH in AGHD patients has a positive impact on BMD, and in some long-term research reports, the replacement of rhGH has significantly improved the BMD of AGHD patients [7–11, 24].

This is an 18-month short-term study. We observed that, compared with the baseline, the BMD of both the lumbar spine and total hip decreased significantly at the 6th month of rhGH replacement therapy, and BMD did not increase or even decreased during the first 12 months of treatment. The first 6 months showed the most significant decline, and no significant change in BMD was observed between the 6th month and the 12th month. At the 18th month, the BMD of the lumbar spine was significantly improved compared with the 6th month and was significantly higher than the baseline at the 18th month. The BMD of the total hip also increased in the 18th month compared with the 6th month, although it was not significant, and after 18 months of treatment, it was still lower than the baseline, but it was not significant. This is consistent with some previous research results. Within 12 months of starting rhGH replacement therapy, BMD cannot be observed to increase or even significantly decrease, and only after 18–24 months can BMD increase be observed. The study of Sartorio [17] showed that BMD during rhGH replacement therapy declined in 1–12 months, with the largest decline in the first 6 months, and bone absorption reached its peak in the 3rd month; Välimäki et al. [30] and Holmes et al. [20] showed that BMD decreased to the lowest point in the sixth month of rhGH replacement therapy and then increased, but no net increase in BMD was observed in 12 months; however, several studies have shown that BMD has a consistent rise in the process of rhGH replacement therapy even in the first 12 months [12, 13, 19].

The serum IGF-1 level of the subjects increased significantly compared with the baseline, indicating that the initial decrease in BMD was not caused by an insufficient increase in IGF-1 levels. The most likely explanation is that GH stimulates bone remodeling [6, 31], a complete bone remodeling cycle is approximately 4 months, and bone resorption occurs before new bone is formed. Therefore, in the initial stage of rhGH replacement therapy, the level of bone resorption is greater than that of bone formation, which is manifested by a decrease in bone density, and it takes 6–12 months for newly formed bone to be completely mineralized. Several studies have shown that rhGH increased the markers of bone turnover between 12 months, reached the peak of bone turnover between the 6th and 10th months, and then decreased, but BMD still showed an upward trend [14, 15, 31]. This provides a good explanation for our research results; rhGH replacement therapy stimulates bone remodeling and increases bone turnover. In the first 6–12 months, it is mainly manifested as bone resorption, and then BMD begins to increase. The deposition and mineralization of new bone takes a certain time, so it lags behind the changes in bone turnover markers. It is also possible that rhGH increases the bone area, which will also affect the results of BMD changes [12, 13, 16, 19]. In general, GH has a positive effect on BMD in patients with AGHD. Although results seen in short-term studies are relatively limited, we can speculate that long-term rhGH replacement therapy should lead to a net increase in BMD, which has also been demonstrated in some long-term studies.

Although the changes we observed in BMD in the lumbar spine and total hip were similar, there were also differences. The change in BMD of the hip was not as significant as that of the lumbar spine, and BMD of the total hip did not change significantly or even decreased slightly from the baseline after 18 months, but BMD of the lumbar spine was significantly higher than the baseline after 18 months of treatment. Our data also show that the percentage change from the baseline in BMD of the lumbar spine was significantly higher than that of the hip after completing 18 months of rhGH replacement therapy, and the BMD of the lumbar spine appeared to be more responsive to GH than that of the hip. This is possibly because the lumbar spine contains more trabecular bone, whereas the femoral neck contains more cortical bone, and trabecular bone is more metabolically active and more susceptible to hormonal influences than cortical bone [20, 32].

We did not observe differences in BMD changes by sex, but we found that patients in the low *Z*-scores' group at the baseline had a greater percentage increase in BMD at both the lumbar spine and total hip than those in the high *Z*-scores' group. The response to GH seems to be more pronounced in patients with low bone mass. Several reports have shown a more pronounced increase in BMD in patients with lower BMD on rhGH replacement therapy, but the differences in BMD changes by gender are controversial. Similar to our results, an 18-month randomized, double-blind, placebo-controlled study of GH replacement in Copenhagen [14] showed no difference in BMD changes between males and females after 18 months of treatment with physiological doses of rhGH replacement in patients with AGHD, while BMD increased more significantly in patients with low Z-scores. In a Finnish multicenter study of rhGH replacement therapy for 42 months [30], the increase in BMD was significantly greater in males than in females, and the percentage increase in BMD was significantly greater in patients with osteopenia at the baseline than in patients with normal bone mass. A 2-year prospective study from Sweden [13] showed that the percentage increase in total body BMD was greater in females than males at the end of rhGH replacement therapy, but no gender differences were observed in BMD changes at other sites, while the percentage increase in BMD was greater in patients with *Z* < −1 at the baseline. A prospective study in the Netherlands [12] of rhGH treatment for two years, in which patients were divided into men, estrogen-replete women, and estrogen-depleted women, showed that changes in *Z*-scores of the lumbar spine did not differ among the three groups, but the study measured only the lumbar spine.

Unfortunately, we did not observe a correlation between IGF-1 levels and BMD at any time point. It may be due to the following reasons: (1) changes in serum IGF-1 are more sensitive to changes in BMD, with a significant increase in IGF-1 during the first month of rhGH treatment, but the changes in BMD are relatively slow and complex, and the formation of new bone takes 6–12 months, so changes in BMD are not concordant with changes in serum IGF-1 levels [3, 6]; (2) the duration of the disease varied in each subject, as did the duration of GH deficiency, IGF-1, which can decrease markedly within a short time after onset, but the decrease in BMD lagged relatively behind; (3) changes in BMD are associated with various factors, such as serum 25-(OH)VD₃ levels, dietary habits, sunlight exposure, and exercise, and failure to use sex hormone replacement therapy in postmenopausal women can also interfere with BMD changes, as can previous hyperpituitarism in some patients with pituitary tumors [33]; (4) although GH acts mainly indirectly through IGF-1, in addition to that, GH can also act directly on bone [23]; (5) the analysis using only the values of IGF-1 did not exclude the interference of age on IGF-1, and the IGF-1 SD score for age should be widely used in the future clinical or scientific research. We also found no correlation between peak GH and BMD or *Z*-scores, and when we grouped BMD and *Z*-scores according to peak GH, no difference was found between the groups. Probably because of the variable course of the disease in each patient, the peak GH could only indicate the degree of GH deficiency present in the patient; however, it was not clear how long the deficient period was, and it was not possible to predict the patients' BMD simply by the peak GH. However, the results of Annamaria, who contradicted our study, divided 101 adult patients with hypopituitarism into very severe GHD, severe GHD, partial GHD, and non-GHD groups according to the peak GH on a GH provocation test and found that in the lumbar spine, the *T*-scores for BMD correlated with the degree of GHD deficiency and that both the peak GH and serum IGF-1 correlated significantly with the *T*-scores [29].

Our data also show that 18 months of rhGH replacement therapy improved body composition. Weight, WC, HC, WHR, and BMI did not change significantly, which is consistent with most long-term studies. However, the effect of rhGH replacement therapy on BMI is controversial, with reports showing an increase in BMI and reports showing no change in BMI [22, 24]. A possible explanation is that the increase in BMI does not necessarily represent an increase in BFM, possibly an increase in LBM and total bone mineral content, but also an increase in body water retention. Patients had significant increases in LBM and total body lean/fat ratio and significant decreases in both TBF% and VAT. However, adipose tissue is not well distinguished from water by DXA, and intracellular water content increases after rhGH replacement therapy. The grip strength of patients was also significantly elevated, which seems to indirectly reflect increased LBM. However, subjects may not have consistent posture when performing grip strength tests and may be affected by developing force in positions such as the shoulder or large arm, and we should standardize their posture in the future grip strength tests. The significant decrease in QoL scores indicates that the patients' quality of life was improved. After patients had passed through rhGH replacement therapy, there was an increase in life well-being and exercise performance, which all helped elevate patients' LBM and BMD, as well as decrease BFM. We also observed a significant decrease in diastolic blood pressure possibly because IGF-1 stimulates the vascular endothelium to produce nitric oxide (NO) to dilate the blood vessels or by increasing the activity of Na/K-ATPase in vascular smooth muscle cells, and it is also possible that rhGH replacement reduced lipid levels [34].

There are several limitations to our study. First, this is a retrospective study, and we cannot have better control of the patients' test indicators: for example, the patients were not tested for serum 25-(OH)VD₃, and we are not clear about specifics such as patients' lifestyle habits and exercise patterns, which are associated with BMD. In addition, our study also lacked a placebo or a control group not treated with rhGH because aging in humans over time counteracts a portion of the effects of rhGH, so the conclusions drawn by relying solely on the pre- and post-treatment controls in the rhGH-treated group are of relatively limited value. Additionally, we only studied the effect of rhGH on BMD in AGHD patients, but it is unclear how GH treatment affects BMD in non-AGHD patients. Prospective controlled studies with large samples are needed in the future to explore the effect of GH on BMD.

## 5. Conclusions

In conclusion, short-term treatment with rhGH replacement for 18 months resulted in beneficial effects on BMD, but these beneficial effects were shown only for the lumbar spine. Although BMD initially decreased, it tended to increase after the 6th month. Meanwhile, both body composition and QoL were improved, which may not only indirectly increase BMD by increasing exercise capacity but also reduce cardiovascular risk. Our study provides some theoretical support for whether rhGH can be used clinically to increase BMD in patients.

## Figures and Tables

**Table 1:** Baseline characteristics of all AGHD patients.

Characteristics	All (*n* = 68)	Males (*n* = 22)	Females (*n* = 46)	*P* value
Age (years)	42.90 ± 11.52	43.55 ± 9.37	42.59 ± 12.51	0.751
Weight (kg)	60.22 ± 10.58	69.74 ± 9.68	55.67 ± 7.58	<0.001^*∗∗*^
Height (cm)	159.88 ± 9.18	168.86 ± 7.10	155.59 ± 6.60	<0.001^*∗∗∗*^
BMI (kg/m^2^)	23.43 ± 2.52	24.40 ± 2.27	22.96 ± 2.52	0.027^*∗*^
Grip strength (kg)	28.39 (21.08, 32.09)	39.28 (32.09, 44.43)	23.18 (20.65, 26.44)	<0.001^*∗∗∗*^
Waist circumference (cm)	83.47 ± 8.88	89.35 ± 7.05	80.65 ± 8.31	<0.001^*∗∗∗*^
Hip circumference (cm)	93.74 (90.35, 98.00)	96.64 (92.88, 99.43)	92.35 (88.15, 99.43)	0.019^*∗*^
WHR	0.89 (0.86, 0.93)	0.92 (0.89, 0.97)	0.87 (0.83, 0.90)	<0.001^*∗∗∗*^
SBP (mm Hg)	113.53 (106.25, 119.00)	118.09 (109.75, 126.50)	111.35 (106.00, 117.50)	0.011^*∗*^
DBP (mm Hg)	75.06 (69.25, 81.00)	79.00 (72.00, 85.00)	73.17 (67.00, 77.25)	0.016^*∗*^
FPG (mmol/L)	5.26 ± 0.44	5.35 ± 0.44	5.21 ± 0.45	0.253
FINS (uIU/ml)	7.33 (4.29, 9.06)	7.94 (2.86, 9.14)	7.03 (4.64, 9.18)	0.723
HbA1c (%)	5.58 (5.40, 5.70)	5.65 (5.50, 5.83)	5.54 (5.40, 5.63)	0.058
TSH (uIU/ml)	2.08 (1.00, 1.73)	2.43 (1.20, 2.78)	1.91 (0.89, 2.58)	0.345
LDL (mmol/L)	2.97 ± 0.91	3.06 ± 1.03	2.92 ± 0.86	0.570
HDL (mmol/L)	1.39 (1.14, 1.60)	1.32 (0.98, 1.51)	1.43 (1.18, 1.60)	0.230
TC (mmol/L)	4.65 ± 1.17	4.78 ± 1.13	4.59 ± 1.2	0.524
TG (mmol/L)	1.50 (0.81, 1.80)	1.45 (0.78, 1.90)	1.52 (0.83, 1.65)	0.704
ALT (U/L)	21.99 (13.00, 28.25)	24.59 (16.25, 31.75)	20.74 (11.75, 25.25)	0.113
AST (U/L)	21.07 (15.00, 26.75)	22.68 (15.00, 27.25)	20.30 (15.00, 22.25)	0.109
Peak GH (ng/ml)	2.11 (0.28, 3.67)	2.00 (0.15, 3.49)	2.15 (0.31, 3.82)	0.704
IGF-1 (ng/ml)	133.63 ± 68.12	137.66 ± 73.99	131.71 ± 65.90	0.739
Lumbar spine BMD (g/cm^2^)	0.860 ± 0.127	0.828 ± 0.108	0.870 ± 0.135	0.323
Total hip BMD (g/cm^2^)	0.820 ± 0.096	0.845 ± 0.101	0.808 ± 0.093	0.139
Lumbar spine *T*-score (SD)	−1.82 ± 1.21	−2.31 ± 0.99	−1.59 ± 1.25	0.022^*∗*^
Lumbar spine *Z*-score (SD)	−1.41 ± 1.18	−2.08 ± 1.05	−1.09 ± 1.10	0.001^*∗∗*^
Total hip *T*-score (SD)	−1.15 ± 0.73	−1.26 ± 0.69	−1.10 ± 0.76	0.413
Total hip *Z*-score (SD)	−0.82 ± 0.73	−1.02 ± 0.75	−0.73 ± 0.71	0.127

SBP, systolic blood pressure; DBP, diastolic blood pressure; TSH, thyroid stimulating hormone; LDL, low-density lipoprotein cholesterol; HDL, high-density lipoprotein cholesterol; TC, total cholesterol; TG, total glyceride; ALT, alanine aminotransferase; AST, aspartate aminotransferase. Data are expressed as the mean ± SD or M (*P*25 and *P*75). ^*∗*^*P* < 0.05 males compared with females; ^*∗∗*^*P* < 0.01 males compared with females; ^*∗∗∗*^*P* < 0.001 males compared with females.

**Table 2:** Changes in BMD during 18 months of rhGH replacement therapy.

	Baseline	6 months	12 months	18 months	6 months vs. baseline	12 months vs. baseline	18 months vs. baseline	18 months vs. 6 months
Lumbar spine	0.860 ± 0.127	0.852 ± 0.127	0.856 ± 0.127	0.871 ± 0.126	*P*=0.006^*∗∗*^	*P*=0.412	*P*=0.045^*∗*^	*P* < 0.001^*∗∗∗*^
Total hip	0.820 ± 0.096	0.815 ± 0.097	0.816 ± 0.097	0.819 ± 0.096	*P*=0.008^*∗∗*^	*P*=0.037^*∗*^	*P*=0.701	*P*=0.201

^*∗*^ *P* < 0.05; ^*∗∗*^*P* < 0.01; ^*∗∗∗*^*P* < 0.001.

**Table 3:** Physical data, body composition, and QoL changes after 18 months of rhGH replacement in 36 patients.

	Baseline	18 months	*P*
Sex (males/females)	7/29	7/29	1
Height (cm)	158.51 ± 8.58	158.55 ± 8.52	0.639
Weight (kg)	58.94 ± 10.47	58.75 ± 9.48	0.694
BMI (kg/m^2^)	23.34 ± 2.67	23.28 ± 2.59	0.754
WC (cm)	81.22 ± 9.47	80.73 ± 9.31	0.404
HC (cm)	93.68 ± 6.59	93.43 ± 6.34	0.635
WHR	0.87 ± 0.65	0.86 ± 0.64	0.502
Grip strength (kg)	26.61 (20.85, 28.25)	28.67 (23.05, 29.6)	0.003^*∗∗*^
SBP (mm Hg)	108.9 ± 10.4	108.5 ± 9.1	0.790
DBP (mm Hg)	73.6 ± 8.0	71.0 ± 5.1	0.036^*∗*^
BFM (g)	19994.3 ± 4652.7	19382.6 ± 4289.4	0.094
LBM (g)	37570.7 (31668.9, 41437.3)	38006.0 (32892.7, 42802.7)	0.012^*∗*^
Body lean/fat ratio	1.97 (1.60, 2.18)	2.06 (1.59, 2.39)	0.003^*∗∗*^
TBF (%)	33.64 (30.6, 37.7)	32.78 (28.5, 37.3)	0.011^*∗*^
VAT (g)	421.89 ± 160.55	393.14 ± 151.70	0.016^*∗*^
QoL (scores)	6.06 (2.00, 10.00)	3.14 (1.00, 3.75)	<0.001^*∗∗∗*^

Data are expressed as the mean ± SD or M (*P*25 and *P*75). ^*∗*^*P* < 0.05; ^*∗∗*^*P* < 0.01; ^*∗∗∗*^*P* < 0.001.

## Data Availability

The data that support the findings of this study are not publicly available for ethical reasons but are available on request.
